# Innate immune mechanisms to oral pathogens in oral mucosa of HIV-infected individuals

**DOI:** 10.1111/odi.13470

**Published:** 2020-09

**Authors:** Aaron Weinberg, Sharof Tugizov, Pushpa Pandiyan, Ge Jin, Srabanti Rakshit, Annapurna Vyakarnam, Julian R. Naglik

**Affiliations:** 1 Department of Biological Sciences, School of Dental Medicine, Case Western Reserve University, Cleveland, OH, USA; 2 Department of Medicine, University of California-San Francisco, San Francisco, CA, USA; 3 Peter Gorer Department of Immunobiology, School of Immunology and Microbial Sciences, Faculty of Life Sciences and Medicine, King’s College London, London, UK; 4 Laboratory of Immunology of HIV-TB co-infection, Centre for Infectious Disease Research, Indian Institute of Science, Bangalore, India; 5 Centre for Host-Microbiome Interactions, Faculty of Dentistry, Oral & Craniofacial Sciences, King’s College London, London, UK

**Keywords:** epithelial, HIV, immunity, innate, oral

## Abstract

A crucial aspect of mucosal HIV transmission is the interaction between HIV, the local environmental milieu and immune cells. The oral mucosa comprises many host cell types including epithelial cells, CD4 + T cells, dendritic cells and monocytes/macrophages, as well as a diverse microbiome predominantly comprising bacterial species. While the oral epithelium is one of the first sites exposed to HIV through oral-genital contact and nursing infants, it is largely thought to be resistant to HIV transmission via mechanisms that are still unclear. HIV-1 infection is also associated with predisposition to secondary infections, such as tuberculosis, and other diseases including cancer. This review addresses the following questions that were discussed at the 8th World Workshop on Oral Health and Disease in AIDS held in Bali, Indonesia, 13 September –15 September 2019: (a) How does HIV infection affect epithelial cell signalling? (b) How does HIV infection affect the production of cytokines and other innate antimicrobial factors, (c) How is the mucosal distribution and function of immune cells altered in HIV infection? (d) How do T cells affect HIV (oral) pathogenesis and cancer? (e) How does HIV infection lead to susceptibility to TB infections?

## 1 | INTRODUCTION

The oral epithelium is a complex structure that provides the first line of innate immune defence against HIV infection (Levy, 2001). It serves as a barrier interface between the external environment (saliva, antimicrobial peptides), resident microbiota and immune cells in the underlying tissues. Studies in primates have long suggested that oral transmission occurs, since oral exposure to SIV results in regional dissemination and subsequent systemic infection (Milush et al., 2004). Therefore, although the oral epithelium may present a barrier to viral transmission, it may also be a conduit for HIV entry. In addition, recent developments in microbiome studies further complicate this complex relationship, as HIV-1 integration/infection of local mucosal epithelial and/or immune cells may predispose individuals to secondary infections and other diseases such as cancer. Currently, our knowledge of the innate immune mechanisms against pathogens in oral mucosa in the context of HIV infection is limited and this workshop aimed to address some of these issues and to identify areas for future research. The workshop questions targeted were as follows:

Question 1. How does HIV infection affect epithelial cell signalling?
Question 2. How does HIV infection affect the production of cytokines and other innate antimicrobial factors.
Question 3. How is the mucosal distribution and function of immune cells altered in HIV infection?
Question 4. How do T cells affect HIV (oral) pathogenesis and cancer?
Question 5. How does HIV infection lead to susceptibility to TB infections?

## 2 | QUESTION 1. HOW DOES HIV INFECTION AFFECT EPITHELIAL CELL SIGNALLING?

### 2.1 | HIV induction of epithelial signalling

Although HIV-activated/modulated multiple signalling pathways in CD4 + T cells, monocytes, macrophages and dendritic cells (DCs) have been intensively investigated, the virus-activated/affected signalling in epithelial cells has been studied mostly with the mitogen-activated protein kinase (MAPK) signalling pathway. The MAPK or Ras-Raf-MEK-ERK signalling pathway is a major pathway that generates multiple signals from cell surface receptors to their target genes for regulation of several cellular functions (Morrison, 2012). HIV envelope protein gp120 and viral transactivator tat may activate MAPK in various epithelial cells, including oral and genital mucosal epithelia, leading to disruption of their tight and adherens junctions. HIV gp120 interacts with galactosylceramide (GalCer) and chemokine receptors CCR5 and/or CXCR4 on the oral, genital and intestinal epithelial surface (Herrera et al., 2016; Kohli et al., 2014; Tugizov et al., 2011; Yasen, Herrera, Rosbe, Lien, & Tugizov, 2018). HIV gp120 binding to GalCer, CCR5 and CXCR4 increases the intracellular calcium concentration, which activates MAPK signalling (Sufiawati & Tugizov, 2014, 2018). This causes the disruption of epithelial junctions by reducing the expression of tight junction proteins ZO-1, occludin and claudins 1, 3 and 4 (Bai et al., 2008; Nazli et al., 2010; Sufiawati & Tugizov, 2014, 2018). Activation of MAPK p38 reduces the expression of occludin and ZO-1 in gastric epithelial cells (Wu et al., 2013). HIV gp120-induced activation of MAPK and disruption of oral and genital mucosal epithelial cells may play a critical role in the paracellular penetration of virus upon primary infection (Nazli et al., 2010), which may initiate the systemic HIV/AIDS disease.

### 2.2 | HIV infection and epithelial tight junctions

In HIV-infected individuals with AIDS, tight junctions in oral, intestinal and genital mucosal epithelia are disrupted (Assimakopoulos, Dimitropoulou, Marangos, & Gogos, 2014; Tugizov et al., 2013). HIV infection is associated with severe impairment of the barrier function of intestinal mucosal epithelium, which leads to diarrhoea and malabsorption (Assimakopoulos et al., 2014; Tugizov et al., 2013). The disruption of intestinal epithelial junctions allows the penetration of commensal gut microbiota and their products into lamina propria and systemic circulation, leading to chronic inflammation and further disruption of gut epithelium, accelerating the progression of HIV/AIDS disease (Vyboh, Jenabian, Mehraj, & Routy, 2015). In HIV-infected individuals, in addition to HIV gp120, the viral transactivator protein tat also induces the disruption of tight junctions. HIV tat contains the tripeptide arginine-glycine-aspartic acid, and its interaction with α5β1, αvβ3 and αvβ3 integrins activates MAPK signalling (Barillari et al., 1999), causing the disruption of tight and adherens junctions of epithelial cells (Sufiawati & Tugizov, 2014, 2018; Tugizov et al., 2013). In retinal pigment epithelial cells, HIV tat induces the activation of ERK1/2 and NF-κB signalling and reduces the expression of claudins 1, 3 and 4 (Bai et al., 2008). Furthermore, in oral epithelial cells HIV gp120 and tat induce the activation of MAPK and the reduction of occludin and E-cadherin expression (Sufiawati & Tugizov, 2014, 2018). The active status of MAPK signalling prevents the association of ZO-1, occludin and claudin-1 with the cell-to-cell contact areas and thus inhibits the formation of tight junctions in epithelial cells (Chen, Lu, Schneeberger, & Goodenough, 2000).

Prolonged interaction of cell-free HIV virions and viral proteins gp120 and tat with oral, cervical and foreskin epithelial cells for 5–7 days leads to constitutive activation of ERK1/2 MAPK and substantial disruption of tight and adherens junctions of oral epithelial cells (Sufiawati & Tugizov, 2014, 2018; Tugizov et al., 2013). Furthermore, such HIV-associated disruption of epithelial junctions initiates the loss of epithelial morphology, followed by the epithelial-mesenchymal transition (EMT). EMT is an epigenetic process that leads to the disruption of mucosal epithelia and allows the paracellular spread of viral and other pathogens. Interaction of cell-free virions and gp120 and tat proteins with epithelial cells substantially reduces E-cadherin expression and activates vimentin and N-cadherin expression, which are well-known mesenchymal markers. HIV gp120-and tat-induced EMT is mediated by SMAD2 phosphorylation and activation of transcription factors Slug, Snail, Twist1 and ZEB1 (Lien, Mayer, Herrera, Rosbe, & Tugizov, 2019). Activation of TGF-β and MAPK signalling by gp120, tat and cell-free HIV virions reveals the critical roles of these signalling pathways in EMT induction. gp120-and tat-induced EMT cells are highly migratory via collagen-coated membranes, which is one of the main features of mesenchymal cells. Thus, if EMT occurs in a premalignant or malignant cell environment of oral and genital mucosa, it may accelerate the neoplastic process by promoting invasion of malignant cells.

It is well known that the expression of proinflammatory cytokines, including TNF-α and IFN-γ, is elevated in the blood and various tissues, including mucosal epithelia, in HIV-infected individuals (Norris et al., 2006; Tugizov, 2016). HIV/AIDS-associated systemic elevation of TNF-α and IFN-γ may activate MAPK, inducing the disruption of epithelial junctions and EMT (Tugizov, 2016).

In summary, HIV/AIDS-associated disruption of oral, intestinal and genital epithelia is still an issue with antiretroviral therapy, possibly because of residual HIV infection of intramucosal CD4 + T lymphocytes, macrophages, Langerhans/dendritic cells and/or HIV/AIDS-initiated chronic inflammation. The identification of critical molecular targets for inhibition of MAPK and TGF-β signalling, chronic inflammation and EMT may lead to the development of efficient drugs that would prevent the disruption of epithelial junctions and preserve their normal barrier functions.

## 3 | QUESTION 2. HOW DOES HIV INFECTION AFFECT THE PRODUCTION OF CYTOKINES AND OTHER INNATE ANTIMICROBIAL FACTORS

### 3.1 | HIV and immunity

To understand how HIV infection affects the production of cytokines and other innate antimicrobial factors, one needs to investigate the dynamics associated with systemic immune activation. In susceptible humans, HIV triggers both the innate and adaptive immune systems (Sauce, Elbim, & Appay, 2013), seemingly to protect the host, but with dire consequences; that is, chronic infection and life-long viral latency that collectively contributes to persistent immune activation and immunodeficiency.

### 3.2 | How does systemic immune activation (SIA) happen?

During the acute phase of an HIV infection, the majority of CD4 + T lymphocytes are lost, with mucosal compartments most severely affected. This is particularly profound in the gut, where the frequency of infection is very high (Brenchley & Douek, 2008). IL-17- and IL-22-producing T cells, which play a role in regulating epithelial homeostasis, decline, thereby compromising gut mucosal integrity. At the same time, neutrophils, monocytes and plasmacytoid DCs (pDCs) accumulate and contribute to an increased inflammatory environment (Brenchley et al., 2008). The pDCs are activated by HIV RNA to release type 1 interferon through activation of toll-like receptors (TLR)7 and 8, leading to suppression of CD4 + T cells in the thymus and induction of CD4 + T-cell apoptosis (Utay & Douek, 2016). The growing acute intestinal inflammation leads to a “leaky gut” owing to alterations in epithelial cell tight junctions along with epithelial cell apoptosis. In the colon, bacterial lipopolysaccharide (LPS) translocates through the epithelial barrier and results in increased circulating LPS levels (Chung, Alden, Funderburg, Fu, & Levine, 2014). The latter correlates with SIA, due to the triggering of inflammation throughout the body. While antiretroviral therapy (ART) promotes CD4 + T-cell reconstitution and lowers plasma LPS levels, weakened intestinal tight junctions persist. This promotes a chronic inflammatory condition, where inflammatory cytokines (e.g. TNF-α, IFN-γ, IL-4, IL-6, IL-10) are found to be significantly higher in intestinal mucosa in HIV subjects despite an increase in CD4 + T cells and a decline in blood and mucosal HIV levels (Schulbin et al., 2008). This gut-centric chronic inflammation appears to be the catalyst for differential acquisition of a wide range of non-communicable complications and diseases including, but not limited to metabolic, cardiovascular, neuro-degenerative, chronic kidney diseases and cancer in people living with HIV (PLWH) (Funderburg et al., 2007).

### 3.3 | What about the oral cavity?

Despite ample evidence that HIV can disrupt tight junctions in oral mucosa and be efficiently transcytosed across epithelial tissues, allowing the virus to gain access to target cells in the lamina propria (Tugizov; see his section in this workshop), rigorous in vivo studies to determine if the oral cavity contributes to SIA are lacking. Instead, emphasis has been placed on predisposition to oral diseases in PLWH. A third to nearly half (32%–46%) of PLWH present with major oral health problems including cancer, ulcers and bacterial, fungal and viral related infections (Li, Kolltveit, Tronstad, & Olsen, 2000), some of which are attributed to long-term use of ART (Nittayananta et al., 2010). This is important considering nine studies suggesting periodontal disease may increase the risk for oral cancer by 2–5-fold (Javed & Warnakulasuriya, 2016). PLWH on ART have a greater risk of oral squamous cell carcinoma (OSCC) of the oropharynx and tongue compared to the general population (Purgina, Pantanowitz, & Seethala, 2011; Tota et al., 2018). Based on these and the published data suggesting a role for ART in the development of non-communicable complications (Casper, Crane, Menon, & Money, 2017), investigators are starting to focus on ART as a possible risk factor for head and neck cancers (HNCs) in PLWH (Silverberg et al., 2015).

We have demonstrated an altered oral mucosal proteome and epigenome in HIV+ on ART subjects versus. HIV-subjects (Ghosh et al., 2013; Yohannes et al., 2011), suggesting that the epithelial barrier may be dysfunctional at multiple levels. Epithelial cells isolated from the oral mucosa of HIV+ on ART subjects grow more slowly, are less innate immune responsive to microbial challenges and have reduced histone deacetylase (HDAC-1) levels and reduced total DNA methyltrasferase activity when compared to oral epithelial cells from age- and sex-matched HIV- subjects. This, along with additional reports showing that oral malignancies are on the rise in PLWH and that ART adversely affects human oral epithelial cells (i.e. abnormal repair/proliferation, telomerase function, DNA synthesis), requires a thorough investigation into the promotion of oral disease associated sequelae through long-term use of ART.

Some have hypothesized that oral microbial dysbiosis may be another risk factor contributing to the oral health disparity experienced by PLWH. PLWH present with a more complex microbial profile in periodontally diseased sites compared with HIV-negative subjects. Organisms such as *Enterococcus faecalis*, *Acinetobacter baumanii*, *Pseudomonas aeruginosa* and *Helicobacter pylori*, which are not usually linked to periodontal disease, have been frequently found in the oral cavities of HIV+ on ART subjects (Goncalves, Souto, & Colombo, 2009). Additionally, ART increases the risk of recovering certain periodontopathogenic bacteria, such as *Fusobacterium* species, *Peptostreptococcus micros*, *Campylobacter species*, *Eubacterium* species and *Tannerella forsythia* (Navazesh et al., 2005). It is intriguing to speculate that the uncommon bacterial species found in PLWH may potentially complex with periodontal species and promote more aggressive oral complications seen in HIV + subjects.

### 3.4 | What about antimicrobial agents?

Over 45 distinct antimicrobial peptides (AMPs) have been identified in human saliva and gingival crevicular fluid (Gorr & Abdolhosseini, 2011). They are part of the innate immune system and provide first line defence against bacteria, fungi and enveloped viruses (Cole & Cole, 2017). They also “cross-talk” with the adaptive immune system as chemokines, adjuvants and ligands for specific host cell receptors. The AMPs most studied with regard to HIV include α- and β-defensins, the cathelicidin LL-37, secretory leukocyte protease inhibitor (SLPI) and calprotectin; although others have been associated with either inhibiting HIV or promoting increased infection, as well as synthetic AMPs being proposed as anti-viral agents (Cole & Cole, 2017).

Infant oral mucosa that does not express human beta defensins (hBDs) is susceptible to infectious HIV transcytosis, while HIV transcytosed across adult oral mucosa, that expresses hBDs, is not infectious (Tugizov et al., 2012). Interestingly, while HIV fails to infect primary human oral epithelial cell monolayers, it induces expression of hBD2 and hBD3 significantly above baseline (Quinones-Mateu et al., 2003). These peptides inhibit HIV-1 replication in immuno-competent cells by binding irreversibly to the virion particles and preferentially downmodulating the CXCR4 co-receptor (Quinones-Mateu et al., 2003). Interestingly, the oral mucosa of HIV + subjects on ART appears to be deficient in hBD2 (Sun et al., 2005), while hBD3 is overexpressed (Zapata et al., 2008). Whether this is due to chronic HIV infection, ART and/or some degree of epithelial cell dysregulation remains an open question.

An interesting example of how HIV hijacks the host’s AMP defenses to promote infection can be found in the example of how genital herpes increases the risk for sexually acquired HIV. Herpes simplex virus (HSV)-2 stimulates expression of epithelial cell-derived hBDs and LL-37. LL-37 increases HIV receptors CD4 and CCR5 on Langerhans cells (LCs), resident DCs and thereby facilitates HIV infection. Additionally, culture supernatants from HSV-2 infected epithelial cells enhance HIV infectivity of LCs, while antibodies directed against LL-37 abrogate infection (Ogawa et al., 2013). While the oral mucosa harbours many cell types that express HIV receptors, including LCs in the epidermis and T cells and macrophages in the lamina propria, oral herpes HSV-1 or infection of the oral cavity with HSV-2 through oral sex, does not appear to predispose the oral mucosa to HIV infection. This may be due, in part to saliva, to the much higher level of hBD expression in the oral mucosa when compared to vaginal mucosa and the more susceptible location of LCs within the vaginal mucosa when compared to its’ oral counterpart.

In summary, it appears that no single AMP is responsible for protecting or endangering the host; that each AMP may act in concert with other substances, and complex interactions are likely. The result may be synergy between several different AMPs produced by the host, where each one individually may not be sufficient to elicit an outcome.

## 4 | QUESTION 3. HOW IS THE MUCOSAL DISTRIBUTION AND FUNCTION OF IMMUNE CELLS ALTERED IN HIV INFECTION?

### 4.1 | HIV and local environmental milieu

Residual oral inflammation in HIV + patients on ART treatment has been linked to a wide range of oral pathologies including opportunistic infections, periodontitis and oral cancer. Although ART can suppress plasma viral loads to undetectable levels, increased mortality is closely associated with mucosal dysbiosis and persistent viral reservoirs in lymphoid tissues in PLWH (Klatt, Funderburg, & Brenchley, 2013). Also, while persistent inflammation in ART-treated PWLH is considered a fallout from gut mucosal catastrophe, dysbiosis, and incomplete restoration of some mucosal immune components, the contribution of oral mucosa is unknown. Given the unique composition of microbes in the oral cavity that is distinct from other mucosa, new information about how microbial products (from altered oral microbiome and opportunistic pathogens) impact persistent oral mucosal inflammation, and function as predisposition factor for HIV-associated tumorigenesis in PWLH remains to be studied (Mukherjee et al., 2018). Microbiome studies in PWLH show alterations in total microbial colonization and composition of the host microbiota in the oral cavity of these individuals. For example, increases in prevalence of several bacterial species such as *Streptococcus mutans*, *Streptococcus sobrinus*, *Lactobacillus species* and the genera *Fusobacterium*, *Campylobacter*, *Prevotella*, *Capnocytophaga*, *Selenomonas*, *Actinomyces*, *Granulicatella* and *Atopobium* were observed in PWLH. While both quantitative and qualitative increase of colonization were observed in some of the bacteria listed above, *Aggregatibacter species* is found at diminished levels in PWLH. Regarding the mycobiome, while the levels of *Candida*, *Aspergillus* and *Cryptococcus species* are elevated, prevalence of the fungus *Pichia* was reduced in ART-treated PWLH. It is intriguing to note that some of the above alterations in oral microbiome correlate with the CD4 T-cell counts in PWLH; however, the details of CD4^+^ T-cell subsets are unknown.

### 4.2 | Immune cells and HIV infection

Recent literature has begun to reveal how microbiome impacts mucosal immune homeostasis by intricately regulating some of the recently characterized T helper cells, such as regulatory T cells (Tregs) and T helper 17 (Th17) cells (Bhaskaran et al., 2018; Pandiyan et al., 2016, 2019). Tregs are CD4^+^CD25^+^FOXP3^+^ cells, which are key immunomodulators with established relevance and therapeutic potential in many disease settings such as infections, cancer, autoimmune diseases and transplantation (Josefowicz, Lu, & Rudensky, 2012; Pandiyan et al., 2011; Pandiyan & Lenardo, 2008; Pandiyan, Zheng, Ishihara, Reed, & Lenardo, 2007; Pandiyan & Zhu, 2015). However, the impact of Tregs on HIV immune pathogenesis in PWLH remains poorly understood (Chevalier et al., 2013). Although initial HIV infection causes depletion of Tregs, leading to lower absolute cell numbers in blood and gut mucosa, increased proportions of Tregs are observed in relation to Th17 cells in gut mucosa. As for Th17 cells, their loss in the gut is linked to loss of mucosal epithelial integrity, and results in deleterious sequelae including microbial translocation and gut inflammation. Preservation of intestinal Th17 cells is critical for their protective effects of limiting intestinal T-cell proliferation/ activation, and microbial translocation in SIV infected rhesus macaques. Incomplete Th17 restoration in the gut despite long-term therapy associates with persistence of immune activation in SIV infected rhesus macaques. While loss of gut mucosal Th17 cells in HIV + individuals lies at the root of gut inflammation and microbial translocation, Th17 and Treg cell functions in oral mucosal tissues and their contribution to persistent inflammation are unclear. Increased gut Treg/Th17 cell ratio correlates to more advanced disease in non-immune responders (CD4 < 350 cells/ml) compared with the HIV controllers and HIV-negative patients (Falivene et al., 2015). Also, increased Treg/Th17 ratio strongly correlates to viral load, plasma levels of sCD14, sCD163 and IL-1RA (markers of monocyte activation), as well as increased T-cell activation (Chevalier et al., 2013; Falivene et al., 2015). However, Tregs’ regulatory function in the context of Treg/Th17 ratio was not examined in these studies (Mendez-Lagares et al., 2012). More importantly, in the immune activation scenario in PWLH, it is not clear whether the increase in ratio of functional Treg/Th17 or the dysfunctional Treg/Th17 cells coincides with disease progression. It is critical to examine Treg functions because immune activation and microbial agonists induce Treg plasticity and trigger the generation of dysfunctional Tregs (Pandiyan & Zhu, 2015). Taken together, while Treg/Th17 alterations may contribute to oral mucosal immune dysregulation during HIV and SIV infection, leading to chronic inflammation and oral cancer, these cells were not studied in PWLH.

A current study by Pandiyan’s group has begun to characterize the immune cell profile in the oral cavity in ART-treated HIV-infected individuals. The study has examined gingival biopsies, saliva and blood correlates to determine the composition and function of immune cells in the context of oral inflammation profile. Initial findings show significant changes in Treg and Th17 cell populations (unpublished, (Pandiyan et al., 2016)). Transcriptomic profiling reveals a significant enrichment of inflammatory and tumorigenesis associated genes in oral mucosa of HIV + patients on ART (unpublished). RNA sequencing and flow cytometry analyses of CD4 + T cells reveals TLR2/Myd88 signalling dysregulation and Treg dysfunction in oral mucosa of HIV + patients on ART, while mechanistic details in the context of dysbiosis remain to be seen. These results warrant further investigations on HIV + oral mucosa answering the following questions; (a) Does oral microbial dysbiosis correlate with TLR2/Myd88 signalling dysregulation? If yes, if this is the cause or consequence for Treg/Th17 dysregulation? (b) What is mechanism of interaction between dysbiotic microbiome and immune cells (c) Which antigen presenting cell or epithelial cell causes the alterations in CD4 + T-cell subsets? (d) Are proinflammatory/anti-inflammatory APC (which engage T cells) differentially regulated in healthy versus HIV + patients? Taken together, the contribution of Th17 and Tregs cells and mechanisms of specific interactions with other immune cells and microbiota in oral mucosa, and answering the pending questions above will be a significant contribution to the biology of oral inflammation of PLWH.

## 5 | QUESTION 4. HOW DO T CELLS AFFECT HIV (ORAL) PATHOGENESIS AND CANCER?

### 5.1 | HIV and cancer

ART has dramatically reduced incidence rates of HIV infection to AIDS. As a result, HIV infection is now a manageable chronic disease and PLWH have similar life spans as the general population. However, as the PLWH population ages, non-AIDS defining cancers, including cancers in the oral cavity/oropharynx, the liver and the lung, become one of the major causes of mortality and morbidity in the population (Goedert, Hosgood, Biggar, Strickler, & Rabkin, 2016; Mahale, Engels, Coghill, Kahn, & Shiels, 2018). HIV-positive individuals with ongoing ART experience immune ageing (immune senescence), including accumulation of CD28^−^/CD57^+^ CD8+ T cells and HLA-DR expression (Appay & Sauce, 2017). Immune senescence of PLWH is associated with the progression of non-AIDS defining cancers and other morbidities, such as kidney disease, diabetes, cardiovascular events and hypertension (Duffau et al., 2015). However, the role of HIV infection in the progression of cancers in PLWH is largely unknown.

### 5.2 | HIV exosomes

Like most cells, HIV-infected T cells secrete a variety of immunologically active extracellular vesicles (EVs, membrane enclosed nanoparticles) to influence intercellular communication and regulate immune response at both local and distant sites, thus potentially contributing to biological outcomes of tumors. Exosomes isolated from culture supernatants of latently HIV-1-infected T-cell lines contain HIV transactivation responsive element (TAR) RNA, and relatively minor amount of Tat and Nef RNA (Chen et al., 2018), indicating that HIV-infected T cells secrete HIV-associated exosomes into the extracellular space. Exosomes are a collected term for EVs of endosomal origin of cells, generated as intraluminal vesicles that bud away from the cytoplasm into an intermediate endocytic compartment termed the multivesicular body (MVB). These vesicles are then shed away from cells upon fusion of the MVB with the plasma membrane (Harding, Heuser, & Stahl, 2013; Tkach & Thery, 2016). Exosomes are sized in the range of 30–200 nm and contain proteins, nucleotides and lipids of cells of origin. Chen et al., 2018 have reported that HIV-associated T-cell exosomes promote proliferation, migration and invasion of human OSCC cells in vitro and enhance the growth of tumors in nude mice inoculated with OSCC cells compared with HIV-negative T-cell exosomes (Chen et al., 2018). They have found that the blood of HIV-infected donors who had ultra-low viral loads with CD4 + T-cell counts over 400 contains HIV-associated exosomes. Interestingly, the plasma from HIV-positive donors promotes the proliferation of OSCC cells compared with that from HIV-negative healthy donors. Importantly, exosomes from the plasma of HIV-positive donors, but not the matched exosome-depleted plasma, stimulate the proliferation of OSCC cells, indicating the presence of tumor-promoting HIV-associated exosomes in the circulation of HIV-infected individuals.

Although the HIV TAR RNA is thought to be delivered into recipient cells via fusion of exosomes with target cell membrane (Sampey et al., 2016), entry of exosomes into recipient cells precedes the binding of exosome membrane protein(s) to cell surface receptors, resulting in structural rearrangement of membranes, lipid reorganization and subsequent entry (Prada & Meldolesi, 2016). Chen et al., 2018 have found that the epidermal growth factor receptor (EGFR) plays a critical role in mediating prompt entry of HIV-associated exosomes. Inhibition of EGFR with Cetuximab, an FDA-approved monoclonal antibody to EGFR for head and neck cancer treatment (Zhang, Saba, Chen, & Shin, 2015), blocked fast entry of exosomes into EGFR-expressing cells and HIV-associated exosome induced OSCC cell proliferation (Chen et al., 2018). Interaction of HIV-associated exosomes with EGFR stimulated phosphorylation of the MAPK ERK1/2, without causing canonical phosphorylation of EGFR in OSCC cells. Since OSCC ubiquitously expresses EGFR, these data suggest that EGFR plays a critical role in mediating OSCC progression in association with HIV-associated exosomes. The research reported by Chen et al., 2018 reveals how HIV-infected cells are involved in the development and progression of oral cancers and the critical roles of the HIV TAR RNA and cancer cell EGFR in the pathological event. Further investigation of the role of TAR RNA-bearing exosomes in the oral pathogenesis in people living with HIV/AIDS is needed.

## 6 | QUESTION 5. HOW DOES HIV INFECTION LEAD TO SUSCEPTIBILITY TO TB INFECTIONS?

### 6.1 | HIV and tuberculosis

**FIGURE 1:**
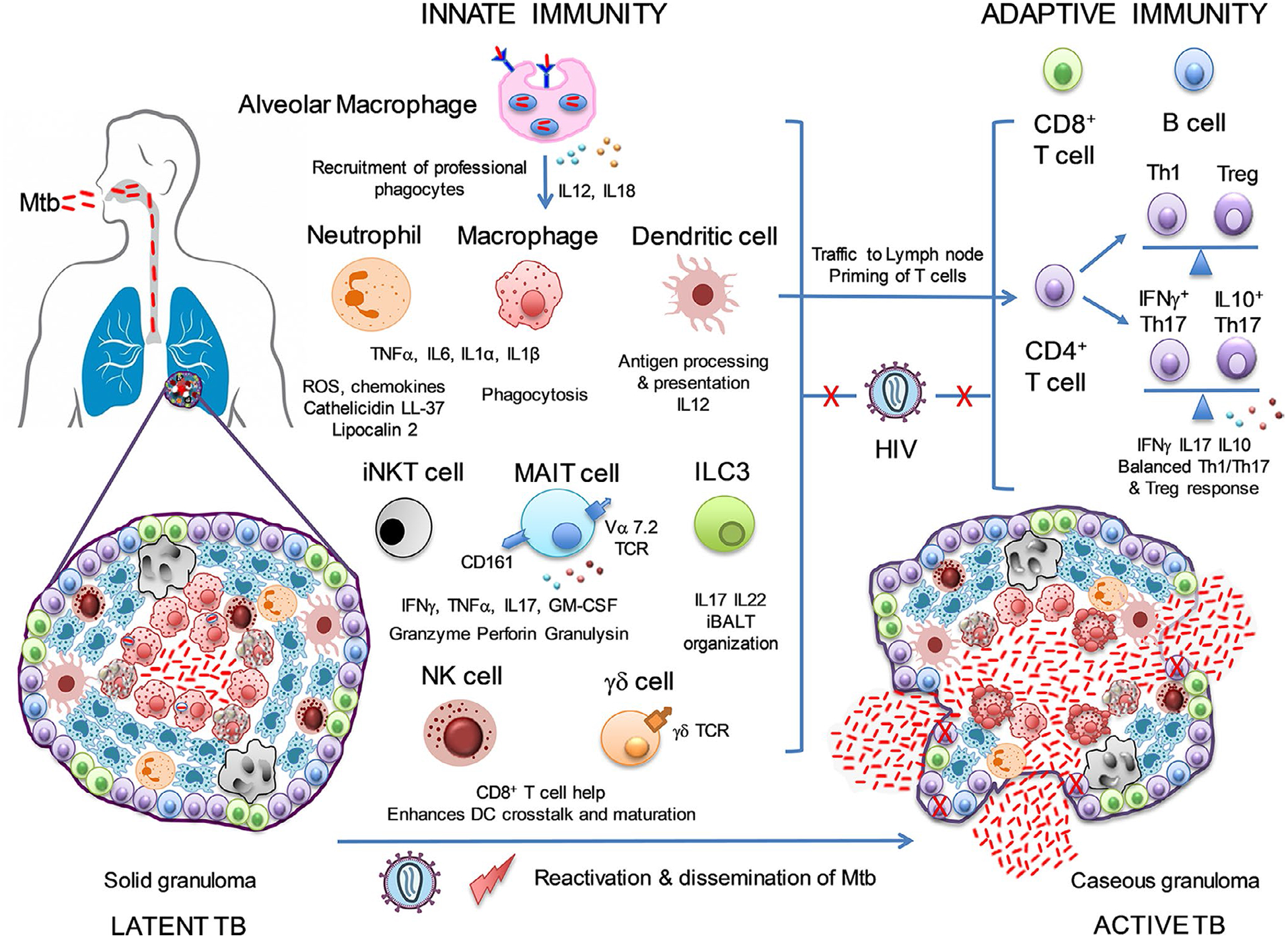
HIV impacts the innate and adaptive immune response that mediates host resistance, clearance of Mtb and leads to reactivation of TB. Alveolar macrophages secrete cytokines upon mycobacterial infection and recruit neutrophils, macrophages and dendritic cells to the lung, which constitute the first line of immune defence and work in a concerted fashion to eliminate Mtb via secretion of proinflammatory cytokines, chemokines, ROS and antimicrobial peptides. Dendritic cells process and present Mtb antigens and traffic to draining lymph nodes to initiate a robust T-cell response. Unconventional T cells like NK, γδ T, MAIT, iNKT-cells and innate lymphoid cells (ILCs) work in a coordinated manner by secretion of cytokines and cytotoxic granules to restrict Mtb growth. CD4^+^ Th1, Th17 and regulatory T (Treg) cells together with CD8^+^ T cells and B cells mount an effective adaptive immune response and maintain a balance between homeostasis and pathogenesis by regulating the secretion of pro- and anti-inflammatory cytokines. HIV leads to the dysregulation of immune responses in the lung and alters with the phagocytic activity of macrophages, inhibits the secretion of cytokines and apoptosis, promotes systemic hyperinflammation and blocks antigen processing and presentation and migration of DCs. HIV also decreases the frequencies of iNKT, MAIT, NK and γδ T cells and interferes with their tissue-homing ability and function. HIV leads to the preferential loss of Mtb-specific CD4^+^ T cells and functional impairment of Th1, Th17 and regulatory T cells, distorts the balanced immune response and skews it towards a proinflammatory profile. Therefore, HIV causes disruption of both innate and adaptive immune responses and predisposes the host to TB

AIDS and tuberculosis (TB) constitute the top 10 leading causes of death worldwide. *Mycobacterium tuberculosis* (Mtb) and HIV, potentiate one another, accelerate the deterioration of host immune responses and augment mortality. In 2018, around one-third of 37.9 million PLWH were estimated to be coinfected with TB and 251,000 people died from HIV-associated TB (WHO, 2019). HIV infection has been established as the single paramount risk factor for reactivation of TB. In fact, the possibility of developing active TB is > 20-fold in HIV-infected than uninfected latent TB subjects; that manifests within the first year of HIV seroconversion, suggesting protective immunity to TB is compromised early after HIV infection. However, despite ART diminishing this risk by 67%, the overall TB incidence rate remained at >4 fold in these subjects (Esmail et al., 2018). Thus, comprehensive understanding of how HIV infection disrupts the network of innate and adaptive immune mechanisms underlying protection and/or clearance of TB infection and alters the balance between immune homeostasis and pathogenesis is of utmost importance (Figure 1).

### 6.2 | Innate immunity to TB: Existing and emerging players

Compelling evidence from in vitro and in vivo studies emphasizes that innate immunity constitutes the first line of defence and impacts the outcome of Mtb infection. Alveolar macrophages induce inflammation by secreting chemokines and recruit professional phagocytes like neutrophils, monocytes and dendritic cells (DC) to the lung, that work in a concerted fashion to eliminate Mtb via production of antimicrobial peptides, inflammatory cytokines and ROS (Gupta, Kumar, & Agrawal, 2018; O’Garra et al., 2013). DCs traffic to lymph nodes to mount robust T-cell activation (O’Garra et al., 2013). Innate immunity also displays adaptive characteristics termed as “trained immunity” as demonstrated by BCG-vaccination, whereby monocytes undergo epigenetic and metabolic reprogramming to promote early Mtb clearance and resistance to subsequent infections by heterologous pathogens (Choreno-Parra, Weinstein, Yunis, Zuniga, & Hernandez-Pando, 2020). Unconventional T cells like NK, γδ-T, MR1-restricted mucosal-associated-invariant-T (MAIT) and invariant-natural-killer-T (iNKT) cells restrict Mtb growth by bridging innate and adaptive immunity (Choreno-Parra et al., 2020; Esmail et al., 2018; Gupta et al., 2018; O’Garra et al., 2013). NK cells upregulate CD8 + T-cell responses by lysis of CD4 + regulatory T cells or Mtb-infected alveolar macrophages in LTBI subjects and enhancing crosstalk with DCs (Choreno-Parra et al., 2020; Gupta et al., 2018). γδ T cells elicit protective immunity through their interaction with NK and CD8 + T cells and maturation of DCs (Gupta et al., 2018; O’Garra et al., 2013). MAIT and iNKT-cells mediate direct Mtb killing via secretion of cytokines (IFNγ/TNFα/IL-17) and cytotoxic molecules (perforin/granulysin/granzyme B) (Esmail et al., 2018; Gupta et al., 2018). Recently, group 3 innate lymphoid cells (ILC3) that functionally resemble CD4^+^ Th17 cells promote the formation of inducible bronchus-associated lymphoid tissue (iBALT) within lung granulomas, by enhancing the production of IL17 and IL-22 and expression of CXCR5 and CXCL13, thus plays crucial role in early control of Mtb (Ardain et al., 2019).

### 6.3 | HIV compromises innate immunity to TB: Fresh perspectives

Enhanced risk of extrapulmonary TB due to HIV infection is linked to neutrophil dysfunction that is associated with defective respiratory burst and phagocytosis and correlates directly with HIV viral load but inversely with CD4 count (Esmail et al., 2018; O’Garra et al., 2013). Indeed, exaggerated inflammation leading to necrotic cell death and enhanced cytotoxic granules release accompanied by distorted immune recovery account for the development of paradoxical TBassociated-immune-reconstitution-inflammatory-syndrome (TB-IRIS) in ART-treated coinfected patients (Esmail et al., 2018). HIV-TB coinfected DCs are defective in antigen-presentation and lead to improper activation of Mtb-specific CD4^+^ T cells. Polymorphisms of DC-SIGN to which HIV-1 gp120 and Mtb-LAM bind, is related to increased susceptibility to TB in HIV + patients (Esmail et al., 2018). HIV depletes CD4^+^ iNKT-cells, thereby eliminating a key innate cell known to eradicate Mtb. Further enhanced frequencies of CD4^−^ cytotoxic iNKT-cells may exacerbate HIV-induced immunopathology in TB-IRIS (Esmail et al., 2018). Paucity of circulating MAIT-cells and their recruitment to the lung is attributed to HIV-driven functional exhaustion and defect in tissue-homing ability of MAIT-cells, characterized by increased PD-1 and decreased CD161 expression, consequently leading to a weakened mucosal immune response that is detrimental to host defence against TB (Esmail et al., 2018). HIV skews the function and phenotype of NK and γδ T cells in coinfected patients and modifies their repertoire leading to TB-IRIS (Esmail et al., 2018; O’Garra et al., 2013). Finally, HIV infection has been reported to decrease the circulating frequencies of ILCs, which given the significance of ILC3s in predicting the outcome of TB, may impact anti-TB immunity (Nabatanzi, Cose, Joloba, Jones, & Nakanjako, 2018).

### 6.4 | Adaptive immunity to TB: Importance of effector and regulatory T cells

Complex interplay of diverse CD4^+^ T cells that act synergistically to establish a well-orchestrated immune response is indispensable for host defence against Mtb infection, as a defective T-cell response leads to persistent infection and failure of resolution of TB. Extrapulmonary Mtb dissemination and maintenance of effector functions of CD8^+^ T and CD3^−^ non-T cells are attributed to CD4^+^ T-cell help (Yao et al., 2014). Th1 cytokine (IFNγ, TNFα, IL12)-deficient mice succumb to Mtb infection due to high bacterial burden, whereas humans undergoing treatment with TNFα-blocking antibodies or having mutations in genes encoding IFNγ, IL-12 or its receptors are susceptible to TB (O’Garra et al., 2013). Beyond Th1 response, Th17 cells have emerged as quintessential players in anti-TB immunity, with the best evidence coming from macaque Mtb-challenge model where mucosal BCG-vaccination induced protection was mediated by Th17 cells (Dijkman et al., 2019). Our data show that a hallmark of latent TB is the presence of IL-10-expressing Th17 cells, the so-called “regulatory Th17” cells, highlighting its potential in anti-TB immunity (Rakshit et al., 2017). Recently, we demonstrated that BCG-revaccination boosts circulating frequencies of this subset (Rakshit et al., 2019). The outcome of Mtb infection also relies on CD4^+^ Tregs that help maintain the delicate balance between pro- and anti-inflammatory CD4^+^ T-cell responses, which has been convincingly demonstrated by the induction of IL-10 and IL-17 in sterile granulomas in macaques (Gideon et al., 2015). Emerging data indicate that this balance is lost in pulmonary TB, primarily due to expansion of activated HLA-DR^+^CD4^+^ T-effectors that acquire resistance to autologous Treg-mediated suppression owing to altered expression of CCR5 and PDL-1 (Ahmed et al., 2018).

### 6.5 | New insights into how HIV compromises adaptive immunity to TB

HIV infection disrupts the global adaptive immune response by depleting CD4^+^ T cells and is one of the foremost factors influencing reactivation of TB in latent Mtb subjects (Esmail et al., 2018; O’Garra et al., 2013). Evidence suggests that HIV predisposes the host to TB by preferential infection and depletion of Mtb-specific CD4^+^ T cells that being CCR5^high^ and MIP-1β^low^ compared with cells of other specifici-ties that are CCR5^low^ and MIP-1β^high^. MIP-1β effectively blocks CCR5 and thereby viral entry (Ahmed, Rakshit, & Vyakarnam, 2016). Whilst the selective depletion of Mtb-specific CD4^+^ T cells has been shown in subjects in the acute phase of HIV infection, our study on latent TB subjects with chronic HIV infection show no evidence for higher viral burden in Mtb-specific cells although, we too report selective loss of Mtb-specific CD4^+^ T cells compared with cells of other specificities, consistent with a chronic SIV-TB coinfection macaque model (Bucsan et al., 2019). What we and others demonstrate is that chronic HIV infection of latent TB subjects has the potential to functionally alter the Mtb-specific CD4^+^ T-cell response by reducing circulating frequencies of Mtb-specific Th1, Th17 polyfunctional cells and inducing expression of activation and exhaustion markers on these cells (Ahmed et al., 2018; Amelio et al., 2019; Rakshit et al., 2017, 2019). We additionally report that HIV has the can skew the Mtb-specific Th17 response by dampening the frequencies of Mtb-specific CD4^+^ Th17 cells that coexpress IL10 without compromising Th17 cells that coexpress IFNγ, thereby inducing a proinflammatory profile, which is associated with immunopathology in other model systems (Bucsan et al., 2019). Thus, HIV-mediated chronic immune activation has the potential to perturb Mtb-specific CD4^+^ T effector cell homeostasis, which is likely crucial for Mtb containment ultimately leading to TB reactivation (Bucsan et al., 2019; Esmail et al., 2018).

In summary, both innate and adaptive arms of anti-TB immunity are severely impacted by HIV. Further studies using whole genome analysis to elucidate the mechanisms by which HIV infection impacts the innate and Mtb-specific adaptive response can help precise dissection of the pathways altered by HIV in subjects with latent TB, thereby predisposing them to TB.

## 7 | FUTURE RESEARCH DIRECTIONS

Several key gaps in our knowledge base were identified in this workshop that may lay the foundation for the future research of innate immune mechanisms against pathogens in oral mucosa of HIV-infected individuals. These include the following:

1. Identification of critical molecular targets for inhibition of MAPK and TGF-β signalling, chronic inflammation and EMT that may lead to the development of efficient drugs to prevent the disruption of epithelial junctions and preserve their normal barrier functions.
2. Determining how to block the key pathways by which epithelial cells transport and deliver HIV virions to susceptible lymphocytes.
3. Identifying the risk factors related to oral mucosal dysfunction in PLWH as the population ages.
4. Detailed investigations are required in exosome research, which is a new area of investigation in the oral arena related to HIV potential risk for dysregulation and promotion of oncogenesis.
5. Correlating oral microbial dysbiosis with Treg/Th17 dysregulation in PLWH and determining whether Treg/Th17 dysregulation in HIV + patients contributes to increased susceptibility of PWLH to oral cancers.
6. Elucidating the mechanisms involved in the HIV-induced switch in the Th17 response, which influences disease outcome and correlates with increased susceptibility to TB.

Oral mucosal dysfunction during HIV infection is highly complex. To truly delineate the critical three-way interaction between the host, the microbiota and the effect of HIV on the system, a real interdisciplinary approach is required that combines detailed biological experimentation with complex systems biology and omics approaches. The future of this area will be interesting to follow.

## References

[R1] Ahmed A, Adiga V, Nayak S, Uday Kumar JAJ, Dhar C, Sahoo PN, … Vyakarnam A (2018). Circulating HLA-DR+CD4+ effector memory T cells resistant to CCR5 and PD-L1 mediated suppression compromise regulatory T cell function in tuberculosis. PLoS Path, 14, e1007289. 10.1371/journal.ppat.1007289 PMC6166982 30231065

[R2] Ahmed A, Rakshit S, & Vyakarnam A (2016). HIV-TB co-infection: Mechanisms that drive reactivation of Mycobacterium tuberculosis in HIV infection. Oral Diseases, 22(Suppl 1), 53–60. 10.1111/odi.12390 27109273

[R3] Amelio P, Portevin D, Hella J, Reither K, Kamwela L, Lweno O, … Perreau M (2019). HIV Infection Functionally Impairs Mycobacterium tuberculosis-Specific CD4 and CD8 T-Cell Responses. Journal of Virology, 93, e01728–e1818. 30541853 10.1128/JVI.01728-18 PMC6384080

[R4] Appay V, & Sauce D (2017). Assessing immune aging in HIV-infected patients. Virulence, 8, 529–538. 27310730 10.1080/21505594.2016.1195536 PMC5538339

[R5] Ardain A, Domingo-Gonzalez R, Das S, Kazer SW, Howard NC, Singh A, … Khader SA (2019). Group 3 innate lymphoid cells mediate early protective immunity against tuberculosis. Nature, 570, 528–532. 31168092 10.1038/s41586-019-1276-2 PMC6626542

[R6] Assimakopoulos SF, Dimitropoulou D, Marangos M, & Gogos CA (2014). Intestinal barrier dysfunction in HIV infection: Pathophysiology, clinical implications and potential therapies. Infection, 42, 951–959. 25070877 10.1007/s15010-014-0666-5

[R7] Bai L, Zhang Z, Zhang H, Li X, Yu Q, Lin H, & Yang W (2008). HIV-1 Tat protein alter the tight junction integrity and function of retinal pigment epithelium: An in vitro study. BMC Infectious Diseases, 8, 77. 18538010 10.1186/1471-2334-8-77 PMC2430207

[R8] Barillari G, Sgadari C, Fiorelli V, Samaniego F, Colombini S, Manzari V, … Ensoli B (1999). The Tat protein of human immunodeficiency virus type-1 promotes vascular cell growth and locomotion by engaging the alpha5beta1 and alphavbeta3 integrins and by mobilizing sequestered basic fibroblast growth factor. Blood, 94, 663–672. 10397733

[R9] Bhaskaran N, Quigley C, Paw C, Butala S, Schneider E, & Pandiyan P (2018). Role of short chain fatty acids in controlling tregs and immunopathology during mucosal infection. Frontiers in Microbiology, 9, 1995. 30197637 10.3389/fmicb.2018.01995 PMC6117408

[R10] Brenchley JM, & Douek DC (2008). HIV infection and the gastrointestinal immune system. Mucosal Immunology, 1, 23–30. 19079157 10.1038/mi.2007.1 PMC2777614

[R11] Brenchley JM, Paiardini M, Knox KS, Asher AI, Cervasi B, Asher TE, … Douek DC (2008). Differential Th17 CD4 T-cell depletion in pathogenic and nonpathogenic lentiviral infections. Blood, 112, 2826–2835. 18664624 10.1182/blood-2008-05-159301 PMC2556618

[R12] Bucsan AN, Chatterjee A, Singh DK, Foreman TW, Lee TH, Threeton B, … Kaushal D (2019). Mechanisms of reactivation of latent tuberculosis infection due to SIV coinfection. J Clin Invest, 129, 5254–5260. 31479428 10.1172/JCI125810 PMC6877319

[R13] Casper C, Crane H, Menon M, & Money D (2017). HIV/AIDS Comorbidities: Impact on Cancer, Noncommunicable Diseases, and Reproductive Health In: Holmes KK, Bertozzi S, Bloom BR, & Jha P eds. Major Infectious Diseases. Washington, DC: The International Bank for Reconstruction and Development/The World Bank (c) 2017 International Bank for Reconstruction and Development/The World Bank. 30212097

[R14] Chen L, Feng Z, Yue H, Bazdar D, Mbonye U, Zender C, … Jin G (2018). Exosomes derived from HIV-1-infected cells promote growth and progression of cancer via HIV TAR RNA. Nature Communications, 9, 4585. 10.1038/s41467-018-07006-2 PMC6214989 30389917

[R15] Chen Y, Lu Q, Schneeberger EE, & Goodenough DA (2000). Restoration of tight junction structure and barrier function by down-regulation of the mitogen-activated protein kinase pathway in ras-transformed Madin-Darby canine kidney cells. Molecular Biology of the Cell, 11, 849–862. 10712504 10.1091/mbc.11.3.849 PMC14815

[R16] Chevalier MF, Petitjean G, Dunyach-Remy C, Didier C, Girard PM, Manea ME, … Weiss L (2013). The Th17/Treg ratio, IL-1RA and sCD14 levels in primary HIV infection predict the T-cell activation set point in the absence of systemic microbial translocation. PLoS Path, 9, e1003453. 10.1371/journal.ppat.1003453 PMC3688532 23818854

[R17] Choreno-Parra JA, Weinstein LI, Yunis EJ, Zuniga J, & Hernandez-Pando R (2020). Thinking outside the box: Innate- and B cell-memory responses as novel protective mechanisms against tuberculosis. Frontiers in Immunology, 11, 226. 32117325 10.3389/fimmu.2020.00226 PMC7034257

[R18] Chung CY, Alden SL, Funderburg NT, Fu P, & Levine AD (2014). Progressive proximal-to-distal reduction in expression of the tight junction complex in colonic epithelium of virally-suppressed HIV+ individuals. PLoS Path, 10, e1004198. 10.1371/journal.ppat.1004198 PMC4072797 24968145

[R19] Cole AM, & Cole AL (2017). HIV-Enhancing and HIV-inhibiting properties of cationic peptides and proteins. Viruses, 9, E108. 28505117 10.3390/v9050108 PMC5454421

[R20] Dijkman K, Sombroek CC, Vervenne RAW, Hofman SO, Boot C, Remarque EJ, … Verreck FAW (2019). Prevention of tuberculosis infection and disease by local BCG in repeatedly exposed rhesus macaques. Nature Medicine, 25, 255–262. 10.1038/s41591-018-0319-9 30664782

[R21] Duffau P, Wittkop L, Lazaro E, le Marec F, Cognet C, Blanco P, … Group ACACS (2015). Association of immune-activation and senescence markers with non-AIDS-defining comorbidities in HIV-suppressed patients. AIDS, 29, 2099–2108. 26544576 10.1097/QAD.0000000000000807

[R22] Esmail H, Riou C, Bruyn ED, Lai RP, Harley YXR, Meintjes G, … Wilkinson RJ (2018). The immune response to mycobacterium tuberculosis in HIV-1-Coinfected persons. Annual Review of Immunology, 36, 603–638. 10.1146/annurev-immunol-042617-053420 29490165

[R23] Falivene J, Ghiglione Y, Laufer N, Eugenia Socias M, Pia Holgado M, Julia Ruiz M, … Magdalena Gherardi M (2015). Th17 and Th17/Treg ratio at early HIV infection associate with protective HIV-specific CD8(+) T-cell responses and disease progression. Scientific Reports, 5, 11511. 26099972 10.1038/srep11511 PMC4477236

[R24] Funderburg N, Lederman MM, Feng Z, Drage MG, Jadlowsky J, Harding CV, … Sieg SF (2007). Human -defensin-3 activates professional antigen-presenting cells via Toll-like receptors 1 and 2. Proceedings of the National Academy of Sciences, 104, 18631–18635. 10.1073/PNAS.0702130104 PMC2141828 18006661

[R25] Ghosh SK, McCormick TS, Eapen BL, Yohannes E, Chance MR, & Weinberg A (2013). Comparison of epigenetic profiles of human oral epithelial cells from HIV-positive (on HAART) and HIV-negative subjects. Epigenetics, 8, 703–709. 23804146 10.4161/epi.25028 PMC3781189

[R26] Gideon HP, Phuah J, Myers AJ, Bryson BD, Rodgers MA, Coleman MT, … Flynn JL (2015). Variability in tuberculosis granuloma T cell responses exists, but a balance of pro- and anti-inflammatory cytokines is associated with sterilization. PLoS Path, 11, e1004603. 10.1371/journal.ppat.1004603 PMC4303275 25611466

[R27] Goedert JJ, Hosgood HD, Biggar RJ, Strickler HD, & Rabkin CS (2016). Screening for cancer in persons living with HIV infection. Trends Cancer, 2, 416–428. 27891533 10.1016/j.trecan.2016.06.007 PMC5120729

[R28] Goncalves LS, Souto R, & Colombo AP (2009). Detection of Helicobacter pylori, Enterococcus faecalis, and Pseudomonas aeruginosa in the subgingival biofilm of HIV-infected subjects undergoing HAART with chronic periodontitis. European Journal of Clinical Microbiology & Infectious Diseases: Official Publication of the European Society of Clinical Microbiology, 28, 1335–1342. 10.1007/s10096-009-0786-5 19639349

[R29] Gorr SU, & Abdolhosseini M (2011). Antimicrobial peptides and periodontal disease. Journal of Clinical Periodontology, 38(Suppl 11), 126–141. 21323710 10.1111/j.1600-051X.2010.01664.x

[R30] Gupta N, Kumar R, & Agrawal B (2018). New players in immunity to tuberculosis: The host microbiome, lung epithelium, and innate immune cells. Frontiers in Immunology, 9, 709. 29692778 10.3389/fimmu.2018.00709 PMC5902499

[R31] Harding CV, Heuser JE, & Stahl PD (2013). Exosomes: Looking back three decades and into the future. Journal of Cell Biology, 200, 367–371. 23420870 10.1083/jcb.201212113 PMC3575527

[R32] Herrera R, Morris M, Rosbe K, Feng Z, Weinberg A, & Tugizov S (2016). Human beta-defensins 2 and −3 cointernalize with human immunodeficiency virus via heparan sulfate proteoglycans and reduce infectivity of intracellular virions in tonsil epithelial cells. Virology, 487, 172–187. 26539799 10.1016/j.virol.2015.09.025 PMC4679645

[R33] Javed F, & Warnakulasuriya S (2016). Is there a relationship between periodontal disease and oral cancer? A systematic review of currently available evidence. Critical Reviews in Oncology/Hematology, 97, 197–205. 26343577 10.1016/j.critrevonc.2015.08.018

[R34] Josefowicz SZ, Lu LF, & Rudensky AY (2012). Regulatory T cells: Mechanisms of differentiation and function. Annual Review of Immunology, 30, 531–564. 10.1146/annurev.immunol.25.022106.141623 PMC6066374 22224781

[R35] Klatt NR, Funderburg NT, & Brenchley JM (2013). Microbial translocation, immune activation, and HIV disease. Trends in Microbiology, 21, 6–13. 23062765 10.1016/j.tim.2012.09.001 PMC3534808

[R36] Kohli A, Islam A, Moyes DL, Murciano C, Shen C, Challacombe SJ, & Naglik JR (2014). Oral and vaginal epithelial cell lines bind and transfer cell-free infectious HIV-1 to permissive cells but are not productively infected. PLoS One, 9, e98077. 24857971 10.1371/journal.pone.0098077 PMC4032250

[R37] Levy JA (2001). The importance of the innate immune system in controlling HIV infection and disease. Trends in Immunology, 22, 312–316. 11377290 10.1016/s1471-4906(01)01925-1

[R38] Li X, Kolltveit KM, Tronstad L, & Olsen I (2000). Systemic diseases caused by oral infection. Clinical Microbiology Reviews, 13, 547–558. 11023956 10.1128/cmr.13.4.547-558.2000 PMC88948

[R39] Lien K, Mayer W, Herrera R, Rosbe K, & Tugizov SM (2019). HIV-1 proteins gp120 and tat induce the epithelial-mesenchymal transition in oral and genital mucosal epithelial cells. PLoS One, 14, e0226343. 31869348 10.1371/journal.pone.0226343 PMC6927651

[R40] Mahale P, Engels EA, Coghill AE, Kahn AR, & Shiels MS (2018). Cancer risk in older people living with human immunodeficiency virus infection in the United States. Clinical Infectious Diseases, 67, 50–57. 29325033 10.1093/cid/ciy012 PMC6248478

[R41] Mendez-Lagares G, Pozo-Balado MM, Genebat M, Garcia Perganeda A, Leal M, & Pacheco YM (2012). Severe immune dysregulation affects CD4(+)CD25(hi)FoxP3(+) regulatory T cells in HIV-infected patients with low-level CD4 T-cell repopulation despite suppressive highly active antiretroviral therapy. Journal of Infectious Diseases, 205, 1501–1509. 22457273 10.1093/infdis/jis230

[R42] Milush JM, Kosub D, Marthas M, Schmidt K, Scott F, Wozniakowski A, … Sodora DL (2004). Rapid dissemination of SIV following oral inoculation. AIDS, 18, 2371–2380. 15622313

[R43] Morrison DK (2012). MAP kinase pathways. Cold Spring Harbor Perspectives in Biology, 4, a011254. 23125017 10.1101/cshperspect.a011254 PMC3536342

[R44] Mukherjee PK, Chandra J, Retuerto M, Tatsuoka C, Ghannoum MA, & McComsey GA (2018). Dysbiosis in the oral bacterial and fungal microbiome of HIV-infected subjects is associated with clinical and immunologic variables of HIV infection. PLoS One, 13, e0200285. 29995962 10.1371/journal.pone.0200285 PMC6040710

[R45] Nabatanzi R, Cose S, Joloba M, Jones SR, & Nakanjako D (2018). Effects of HIV infection and ART on phenotype and function of circulating monocytes, natural killer, and innate lymphoid cells. AIDS Research and Therapy, 15, 7. 29544508 10.1186/s12981-018-0194-y PMC5853105

[R46] Navazesh M, Mulligan R, Pogoda J, Greenspan D, Alves M, Phelan J, … Slots J (2005). The effect of HAART on salivary microbiota in the Women’s Interagency HIV Study (WIHS). Oral Surgery, Oral Medicine, Oral Pathology, Oral Radiology, and Endodontics, 100, 701–708. 10.1016/j.tripleo.2004.10.011 16301151

[R47] Nazli A, Chan O, Dobson-Belaire WN, Ouellet M, Tremblay MJ, Gray-Owen SD, … Kaushic C (2010). Exposure to HIV-1 directly impairs mucosal epithelial barrier integrity allowing microbial translocation. PLoS Path, 6, e1000852. 10.1371/journal.ppat.1000852 PMC2851733 20386714

[R48] Nittayananta W, Talungchit S, Jaruratanasirikul S, Silpapojakul K, Chayakul P, Nilmanat A, & Pruphetkaew N (2010). Effects of long-term use of HAART on oral health status of HIV-infected subjects. Journal of Oral Pathology and Medicine, 39, 397–406. 20202089 10.1111/j.1600-0714.2009.00875.x PMC3217232

[R49] Norris PJ, Pappalardo BL, Custer B, Spotts G, Hecht FM, & Busch MP (2006). Elevations in IL-10, TNF-alpha, and IFN-gamma from the earliest point of HIV Type 1 infection. AIDS Research and Human Retroviruses, 22, 757–762. 16910831 10.1089/aid.2006.22.757 PMC2431151

[R50] O’Garra A, Redford PS, McNab FW, Bloom CI, Wilkinson RJ, & Berry MP (2013). The immune response in tuberculosis. Annual Review of Immunology, 31, 475–527. 10.1146/annurev-immunol-032712-095939 23516984

[R51] Ogawa Y, Kawamura T, Matsuzawa T, Aoki R, Gee P, Yamashita A, … Shimada S (2013). Antimicrobial peptide LL-37 produced by HSV-2-infected keratinocytes enhances HIV infection of Langerhans cells. Cell Host & Microbe, 13, 77–86. 23332157 10.1016/j.chom.2012.12.002

[R52] Pandiyan P, Bhaskaran N, Zou M, Schneider E, Jayaraman S, & Huehn J (2019). Microbiome dependent regulation of Tregs and Th17 cells in mucosa. Frontiers in Immunology, 10, 426. 30906299 10.3389/fimmu.2019.00426 PMC6419713

[R53] Pandiyan P, Conti HR, Zheng L, Peterson AC, Mathern DR, Hernandez-Santos N, … Lenardo MJ (2011). CD4(+)CD25(+) Foxp3(+) regulatory T cells promote Th17 cells in vitro and enhance host resistance in mouse Candida albicans Th17 cell infection model. Immunity, 34, 422–434. 21435589 10.1016/j.immuni.2011.03.002 PMC3258585

[R54] Pandiyan P, & Lenardo MJ (2008). The control of CD4+CD25+Foxp3+ regulatory T cell survival. Biology Direct, 3, 6. 18304352 10.1186/1745-6150-3-6 PMC2270257

[R55] Pandiyan P, Younes S, Ribeiro S, Talla A, Bhaskaran N, McDonald D, … Sekaly R (2016). Mucosal regulatory T cells and T helper 17 cells in HIV associated immune activation. Frontiers in Immunology, 7, 228. 27379092 10.3389/fimmu.2016.00228 PMC4913236

[R56] Pandiyan P, Zheng L, Ishihara S, Reed J, & Lenardo MJ (2007). CD4(+)CD25(+)Foxp3(+) regulatory T cells induce cytokine deprivation-mediated apoptosis of effector CD4(+) T cells. Nature Immunology, 8, 1353–1362. 17982458 10.1038/ni1536

[R57] Pandiyan P, & Zhu J (2015). Origin and functions of pro-inflammatory cytokine producing Foxp3(+) regulatory T cells. Cytokine, 76, 13–24. 26165923 10.1016/j.cyto.2015.07.005 PMC4969074

[R58] Prada I, & Meldolesi J (2016). Binding and Fusion of Extracellular Vesicles to the Plasma Membrane of Their Cell Targets. International Journal of Molecular Sciences, 17, E1296. 27517914 10.3390/ijms17081296 PMC5000693

[R59] Purgina B, Pantanowitz L, & Seethala RR (2011). A review of carcinomas arising in the head and neck region in HIV-positive patients. Pathology Research International, 2011, 469150. 10.4061/2011/469150 PMC3108450 21660273

[R60] Quinones-Mateu ME, Lederman MM, Feng Z, Chakraborty B, Weber J, Rangel HR, … Weinberg A (2003). Human epithelial beta-defensins 2 and 3 inhibit HIV-1 replication. AIDS, 17, F39–F48. 14571200 10.1097/00002030-200311070-00001

[R61] Rakshit S, Adiga V, Nayak S, Sahoo PN, Sharma PK, van Meijgaarden KE, … Vyakarnam A (2017). Circulating Mycobacterium tuberculosis DosR latency antigen-specific, polyfunctional, regulatory IL10(+) Th17 CD4 T-cells differentiate latent from active tuberculosis. Scientific Reports, 7, 11948. 28931830 10.1038/s41598-017-10773-5 PMC5607261

[R62] Rakshit S, Ahmed A, Adiga V, Sundararaj BK, Sahoo PN, Kenneth J, … Vyakarnam A (2019). BCG revaccination boosts adaptive polyfunctional Th1/Th17 and innate effectors in IGRA+ and IGRA- Indian adults. JCI Insight, 4, 130540. 10.1172/jci.insight.130540 PMC6975271 31743110

[R63] Sampey GC, Saifuddin M, Schwab A, Barclay R, Punya S, Chung MC, … Kashanchi F (2016). Exosomes from HIV-1-infected cells stimulate production of pro-inflammatory cytokines through trans-activating response (TAR) RNA. The Journal of Biological Chemistry, 291, 1251–1266. 26553869 10.1074/jbc.M115.662171 PMC4714213

[R64] Sauce D, Elbim C, & Appay V (2013). Monitoring cellular immune markers in HIV infection: From activation to exhaustion. Current Opinion in HIV and AIDS, 8, 125–131. 23380653 10.1097/COH.0b013e32835d08a9

[R65] Schulbin H, Bode H, Stocker H, Schmidt W, Zippel T, Loddenkemper C, … Ullrich R (2008). Cytokine expression in the colonic mucosa of human immunodeficiency virus-infected individuals before and during 9 months of antiretroviral therapy. Antimicrobial Agents and Chemotherapy, 52, 3377–3384. 18573939 10.1128/AAC.00250-08 PMC2533489

[R66] Silverberg MJ, Lau B, Achenbach CJ, Jing Y, Althoff KN, D’Souza G, … Dubrow R (2015). Cumulative incidence of cancer among persons with HIV in North America: A cohort study. Annals of Internal Medicine, 163, 507–518. 26436616 10.7326/M14-2768 PMC4711936

[R67] Sufiawati I, & Tugizov SM (2014). HIV-Associated Disruption of Tight and Adherens Junctions of Oral Epithelial Cells Facilitates HSV-1 Infection and Spread. PLoS One, 9, e88803. 24586397 10.1371/journal.pone.0088803 PMC3931628

[R68] Sufiawati I, & Tugizov SM (2018). HIV-induced matrix metalloproteinase-9 activation through mitogen-activated protein kinase signalling promotes HSV-1 cell-to-cell spread in oral epithelial cells. Journal of General Virology, 99, 937–947. 29775175 10.1099/jgv.0.001075 PMC6537617

[R69] Sun L, Finnegan CM, Kish-Catalone T, Blumenthal R, Garzino-Demo P, La Terra Maggiore GM, … Garzino-Demo A (2005). Human beta-defensins suppress human immunodeficiency virus infection: Potential role in mucosal protection. Journal of Virology, 79, 14318–14329. 16254366 10.1128/JVI.79.22.14318-14329.2005 PMC1280242

[R70] Tkach M, & Thery C (2016). Communication by extracellular vesicles: Where we are and where we need to go. Cell, 164, 1226–1232. 26967288 10.1016/j.cell.2016.01.043

[R71] Tota JE, Engels EA, Madeleine MM, Clarke CA, Lynch CF, Ortiz AP, … Chaturvedi AK (2018). Risk of oral tongue cancer among immunocompromised transplant recipients and human immunodeficiency virus-infected individuals in the United States. Cancer, 124, 2515–2522. 29645080 10.1002/cncr.31359

[R72] Tugizov S (2016). Human immunodeficiency virus-associated disruption of mucosal barriers and its role in HIV transmission and pathogenesis of HIV/AIDS disease. Tissue Barriers, 4, e1159276. 27583187 10.1080/21688370.2016.1159276 PMC4993574

[R73] Tugizov SM, Herrera R, Chin-Hong P, Veluppillai P, Greenspan D, Michael Berry J, … Palefsky JM (2013). HIV-associated disruption of mucosal epithelium facilitates paracellular penetration by human papillomavirus. Virology, 446, 378–388. 24074602 10.1016/j.virol.2013.08.018 PMC3809142

[R74] Tugizov SM, Herrera R, Veluppillai P, Greenspan D, Soros V, Greene WC, … Palefsky JM (2011). HIV is inactivated after transepithelial migration via adult oral epithelial cells but not fetal epithelial cells. Virology, 409, 211–222. 21056450 10.1016/j.virol.2010.10.004 PMC3034249

[R75] Tugizov SM, Herrera R, Veluppillai P, Greenspan D, Soros V, Greene WC, … Palefsky JM (2012). Differential transmission of HIV traversing fetal oral/intestinal epithelia and adult oral epithelia. Journal of Virology, 86, 2556–2570. 22205732 10.1128/JVI.06578-11 PMC3302289

[R76] Utay NS, & Douek DC (2016). Interferons and HIV infection: The good, the bad, and the ugly. Pathogens & Immunity, 1, 107–116. 27500281 10.20411/pai.v1i1.125 PMC4972494

[R77] Vyboh K, Jenabian MA, Mehraj V, & Routy JP (2015). HIV and the gut microbiota, partners in crime: Breaking the vicious cycle to un-earth new therapeutic targets. Journal of Immunology Research, 2015, 614127. 10.1155/2015/614127 PMC4352503 25759844

[R78] World Health Organization (WHO) (2019). HIV factsheets 2019. Retrieved from http://www.who.int/news-room/fact-sheets/detail/hiv-aids. Date last updated: 25 November 2019. Date last accessed: March 31, 2020

[R79] Wu HL, Gao X, Jiang ZD, Duan ZT, Wang SK, He BS, … Xie HG (2013). Attenuated expression of the tight junction proteins is involved in clopidogrel-induced gastric injury through p38 MAPK activation. Toxicology, 304, 41–48. 23220562 10.1016/j.tox.2012.11.020

[R80] Yao S, Huang D, Chen CY, Halliday L, Wang RC, & Chen ZW (2014). CD4+ T cells contain early extrapulmonary tuberculosis (TB) dissemination and rapid TB progression and sustain multieffector functions of CD8+ T and CD3- lymphocytes: Mechanisms of CD4+ T cell immunity. Journal of Immunology (Baltimore, Md.: 1950), 192, 2120–2132. 10.4049/jimmunol.1301373 PMC4104690 24489088

[R81] Yasen A, Herrera R, Rosbe K, Lien K, & Tugizov SM (2018). HIV internalization into oral and genital epithelial cells by endocytosis and macropinocytosis leads to viral sequestration in the vesicles. Virology, 515, 92–107. 29277006 10.1016/j.virol.2017.12.012 PMC5823522

[R82] Yohannes E, Ghosh SK, Jiang B, McCormick TS, Weinberg A, Hill E, … Chance MR (2011). Proteomic signatures of human oral epithelial cells in HIV-infected subjects. PLoS One, 6, e27816. 22114700 10.1371/journal.pone.0027816 PMC3218055

[R83] Zapata W, Rodriguez B, Weber J, Estrada H, Quinones-Mateu ME, Zimermman PA, … Rugeles MT (2008). Increased levels of human beta-defensins mRNA in sexually HIV-1 exposed but uninfected individuals. Current HIV Research, 6, 531–538. 18991618 10.2174/157016208786501463 PMC4126611

[R84] Zhang J, Saba NF, Chen GZ, & Shin DM (2015). Targeting HER (ERBB) signaling in head and neck cancer: An essential update. Molecular Aspects of Medicine, 45, 74–86. 26163475 10.1016/j.mam.2015.07.001 PMC8028037

